# Load displacement and high speed nanoindentation data set at different state of charge (SoC) for spinel Li*_x_*Mn_2_O_4_ cathodes

**DOI:** 10.1016/j.dib.2016.05.034

**Published:** 2016-05-24

**Authors:** Muhammad Zeeshan Mughal, Riccardo Moscatelli, Marco Sebastiani

**Affiliations:** Engineering Department, “Roma TRE” University, Via della Vasca Navale 79, 00146 Rome, Italy

**Keywords:** Fracture toughness, Pillar, Lithium ion battery, Nanoindentation, State of charge

## Abstract

Novel high speed nanoindentation data is reported for 0% and 100% state of charge (SoC) for the spinal Li*_x_*Mn_2_O_4_ material. The article also includes the load/displacement data for different SoC highlighting the displacement bursts corresponding to the pillar splitting for fracture toughness evaluation. For more details, please see the article; Mughal et al. (2016) [1].

**Specifications Table**

**Table:** 

Subject area	*Physics*
More specific subject area	*Nanoindentation*
Type of data	*Tables, figures*
How data was acquired	*Nanoindentation; G*200 *from keysight technologies*
Data format	*Raw, filtered, analyzed, etc.*
Experimental factors	*Commercially available lithium-ion battery cathode materials are used according to industrial standards with the thickness of* 150 *µm and the typical particle radius of* 10 *µm. The sample sections were embedded in a commercial epoxy for mechanical stability during polishing and indenting. Detail description on battery opening and sample preparation is available in Ref.*[2]. 3000 *indentations were performed in less than an hour with the penetration depth of* 100 *nm using high speed nanoindentation with standard Berkovich tip (“Express Test” nanoindentation option provided by Keysight technologies). Focused ion beam (FIB) milled micro pillars were tested using conventional nanoindentation by employing a standard Berkovich tip at a strain rate of* 0.05 *s*^−1^.
Experimental features	*For each sample, experimental modulus was evaluated by statistical deconvolution of the* 3000 *performed tests, without filtering because of the high signal to noise ratio during high speed nanoindentation. Statistical deconvolution was performed according to the recently published method*[2], [3]. *Pillar splitting load was identified by the displacement bursts on a load displacement curve.*
Data source location	*Interdepartmental Laboratory of Electron Microscopy (LIME) of University of “Roma TRE”, Rome, Italy.*
Data accessibility	*Data is with this article*

**Value of the data**

• The data of the load displacement curves obtained during pillar indentation can be used to directly calculate toughness of the materials.
• The data from high-speed nanoindentation can be useful to generate modulus maps in the heterogeneous battery composite under investigation, and then evaluate the single phases.
• The provided data is extremely useful to understand the microstructure-property-performance correlation functions in Lithium-battery composites.

## 1 Data

Experimental data of the high speed nanoindentation for 900 indentations performed in the shape of a matrix along with the load displacement curves for the focused ion beam (FIB) milled pillar splitting. Load displacement nanoindentation curves related to pillar splitting experiments, to evaluate fracture toughness as a function of lithiation.

## 2 Experimental design, materials and methods

**Fig. 1:**
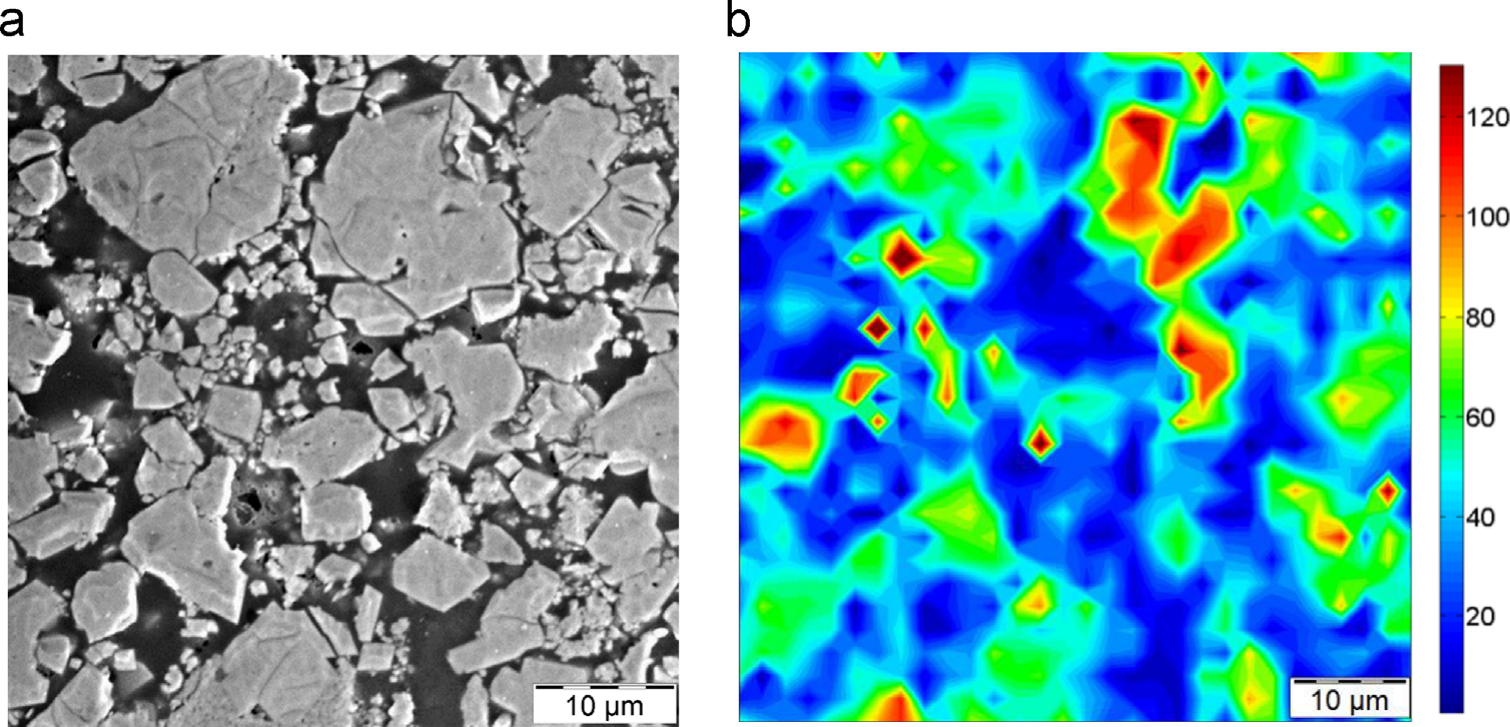
(a) SEM micrograph highlighting the nanoindentation area for Li*_x_*Mn_2_O_4_ cathode and (b) 2D nanoindentation modulus maps (units in GPa).

More than 3000 valid measurements with a penetration depth of 100 nm were performed using high-speed nanoindentation mapping in less than one hour on strongly in-homogeneous battery composites using the G200 Keysight nanoindenter equipped with express-test option. The statistical deconvolution on the cumulative distributions functions of hardness and elastic modulus are performed, according to a procedure that was recently published by the authors [2], [3]. No filtering tools were required in this case, because of the higher signal-to-noise ratio of the high-speed data, which allows for determination of all mechanical phases without filtering of the data, in comparison with the standard tests. Fig. 1, highlights the SEM micrographs of a Li*_x_*Mn_2_O_4_ cathode material along with the 2D nanoindentation map.

For more details please see ref. [1].

**Fig. 2:**
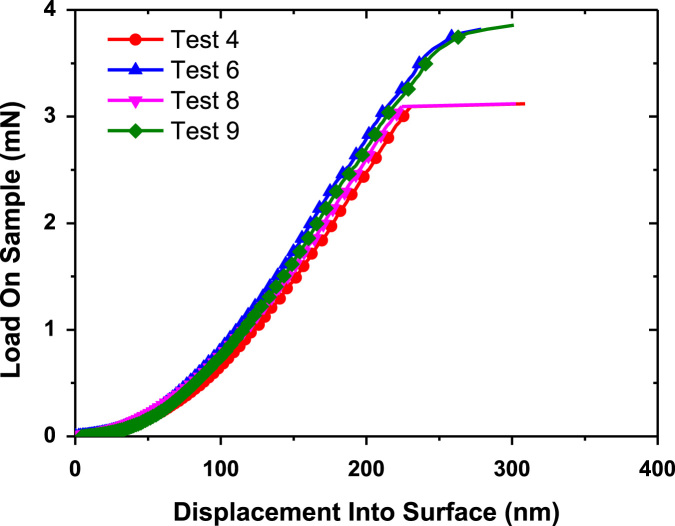
Representative Load displacement curves of Li*_x_*Mn_2_O_4_ cathode material.

Pillar nanoindentation was performed with the help of G200 system from Keysight technologies by employing a XP indentation head at a strain rate of 0.05 s^−1^. Detail description about the modelling activities and application of the pillar splitting technique can be found in previous publications [4], [5]. All indentation tests were performed using a calibrated Berkovich indenter with a maximum indentation depth of 2000 nm. The instrument frame stiffness and the indentation area were calibrated before and after the experiment on a certified fused silica reference sample. The continuous stiffness measurement (CSM) mode was switched off during the tests. The load displacement curve data can be found in the attached excel sheet while Fig. 2 highlights the representative load–displacement curve highlighting the displacement bursts for Li*_x_*Mn_2_O_4_ cathode material.

Load-displacement curves corresponding to different SoC can be found in ref. [1].
