# Supplementary material for: Load displacement and high speed nanoindentation data set at different state of charge (SoC) for spinel LixMn2O4 cathodes
Source: Data Brief. 2016 May 24;8:203–6. doi: 10.1016/j.dib.2016.05.034 (PMC4898909; doi:10.1016/j.dib.2016.05.034)
Supplement: Supplementary material [file mmc1.pdf]

## Section 1. Identifying Information

1. Given Name (First Name)
Marco

2. Surname (Last Name)
Sebastiani

3. Date
08-April-2016

4. Are you the corresponding author? ☑ Yes ☐ No

5. Manuscript Title
Load Displacement and high speed nanoindentation data set at different state of charge (SoC) for spinel LixMn2O4 cathodes

6. Manuscript Identifying Number (if you know it)
DIB-D-16-00073

## Section 2. The Work Under Consideration for Publication

Did you or your institution **at any time** receive payment or services from a third party (government, commercial, private foundation, etc.) for any aspect of the submitted work (including but not limited to grants, data monitoring board, study design, manuscript preparation, statistical analysis, etc.)?

Are there any relevant conflicts of interest? ☐ Yes ☑ No

## Section 3. Relevant financial activities outside the submitted work.

Place a check in the appropriate boxes in the table to indicate whether you have financial relationships (regardless of amount of compensation) with entities as described in the instructions. Use one line for each entity; add as many lines as you need by clicking the "Add +" box. You should report relationships that were **present during the 36 months prior to publication**.

Are there any relevant conflicts of interest? ☐ Yes ☑ No

## Section 4. Intellectual Property -- Patents & Copyrights

Do you have any patents, whether planned, pending or issued, broadly relevant to the work? ☐ Yes ☑ No

## Section 5. Relationships not covered above

Are there other relationships or activities that readers could perceive to have influenced, or that give the appearance of potentially influencing, what you wrote in the submitted work?

☐ Yes, the following relationships/conditions/circumstances are present (explain below):

☑ No other relationships/conditions/circumstances that present a potential conflict of interest

At the time of manuscript acceptance, journals will ask authors to confirm and, if necessary, update their disclosure statements. On occasion, journals may ask authors to disclose further information about reported relationships.

## Section 6. Disclosure Statement

Based on the above disclosures, this form will automatically generate a disclosure statement, which will appear in the box below.

Dr. Sebastiani has nothing to disclose.

## Evaluation and Feedback

Please visit http://www.icmje.org/cgi-bin/feedback to provide feedback on your experience with completing this form.



Marco Sebastiani
